# Glucose metabolism and its direct action in cancer and immune regulation: opportunities and challenges for metabolic targeting

**DOI:** 10.1186/s12929-025-01167-1

**Published:** 2025-07-29

**Authors:** Bo-Syong Pan, Che-Chia Hsu, Hsin-En Wu, Yuan-Ru Chen, Xiaobo Zhou, Shu-Chi Wang, Chia-Yang Li, Hui-Kuan Lin

**Affiliations:** 1 Department of Pathology, Duke University Medical Center, Duke University School of Medicine, Durham, NC 27710 USA; 2 Center for Computational Systems Medicine, McWilliams School of Biomedical Informatics, The University of Texas Health Science Center at Houston, Houston, TX 77030 USA

**Keywords:** Glucose metabolism, Warburg effect, Tumor microenvironment, Immune regulation, Glucose sensor, Immunotherapy resistance, Metabolic targeting, Cancer therapy

## Abstract

Glucose metabolism is a pivotal hub for cellular energy production and the generation of building blocks that support cell growth, survival, and differentiation. Cancer cells undergo metabolic reprogramming to sustain rapid proliferation, survive in harsh microenvironments, and resist therapies. Beyond producing energy and building blocks to meet cancer cell demands, glucose metabolism generates numerous metabolites that serve as signaling molecules, orchestrating signaling pathways and epigenetic modifications that regulate cancer cell phenotypes and immunity. In this review, we discuss how glucose, through its metabolism and direct actions, influences diverse biological processes driving cancer progression and therapeutic resistance, while also exploring metabolic vulnerabilities in cancer for therapeutic strategies.

## Introduction

Glucose metabolism is a fundamental cellular process that sustains energy production, biosynthetic demands, and signaling cascades critical for both normal physiology and pathological states like cancer. In normal cells, glucose is metabolized through glycolysis to pyruvate, which is transported to the mitochondrial for tricarboxylic acid (TCA) cycle entry that supports oxidative phosphorylation for efficient energy production to maintain cell proliferation and survival [1]. In addition, glucose-6-phosphoate (G6P) generated from the first step of glycolysis can shunt into the pentose phosphate pathway (PPP) to generate ribose-5-phosphate (R5P) for nucleotide synthesis and nicotinamide adenine dinucleotide phosphate (NADPH) for redox balance to meet cell proliferation and survival [2]. These pathways are tightly regulated to maintain cellular needs, with gluconeogenesis that synthesizes glucose from non-carbohydrate precursors like lactate and amino acids during nutrient scarcity [3].

In cancer, glucose metabolism undergoes profound reprogramming to meet the heightened demands of rapidly dividing cells, a phenomenon first described by Otto Warburg as the Warburg effect [4]. Cancer cells preferentially utilize aerobic glycolysis to support rapid ATP production and the intermediates critical for the biosynthesis of nucleotides, lipids, and amino acids by converting glucose to lactate even in oxygen-rich conditions [5]. This metabolic reprogramming can be driven by numerous oncogenes, such as MYC, RAS, and hypoxia-inducible factor-1α (HIF-1α), which upregulate glucose transporters (GLUT1 and GLUT3) and glycolytic enzymes including hexokinase 2 (HK2), pyruvate kinase M2 (PKM2) and lactate dehydrogenase A (LDHA) to enhance glucose uptake and flux [6]. Beyond energy production and biosynthesis, glucose-derived metabolites such as lactate, inositol, fructose-1,6-bisphosphate (FBP), phosphoenolpyruvate (PEP), α-ketoglutarate (α-KG), and 2-hydroxyglutarate (2HG, included D-2HG and L-2HG) shape the tumor microenvironment (TME), promoting immunosuppression by inhibiting cytotoxic T cells and inducing M2-like macrophage polarization [7].

Immune cells also rely on glucose metabolism, with effector T cells, M1 macrophages, and activated dendritic cells (DCs) adopting glycolysis to fuel proliferation and cytokine production, while regulatory T cells (Tregs) and M2 macrophages favor oxidative phosphorylation (OXPHOS) [8]. This metabolic convergence between cancer and immune cells creates competition for glucose in the TME, where tumors often outcompete immune cells, impairing anti-tumor immunity [9]. Additionally, glucose-derived metabolites and oncometabolites act as signaling molecules, altering epigenetic landscapes and immune responses through histone modifications and protein succination [10].

While glucose metabolism plays a critical role for regulating cancer and immune phenotypes, recent studies have unveiled glucose’s non-metabolic roles as a signaling molecule in regulating oncogenic and immune pathways [11, 12]. These findings highlight the multifaceted roles of glucose in cancer and immunity. In this review, we will dissect how glucose acts through its metabolism and direct actions to orchestrate cancer progression, immune regulation, and therapeutic resistance, explore therapeutic strategies by targeting metabolic vulnerabilities in cancer, and finally address challenges, such as metabolic plasticity and tissue specificity.

## Part 1: glucose metabolism in cancer and immune regulation

### Aerobic glycolysis

Cancer cells autonomously alter metabolic pathways to meet the bioenergetic and biosynthetic demands of proliferation and survival. Under distinct physiological or pathological conditions, malignant cells are addicted to utilizing nutrient metabolism and signaling pathways distinct from those of non-malignant cells. Dr. Otto Warburg discovered that numerous tumors rely on aerobic glycolysis, known as the Warburg effect, to convert most glucose to lactate, a prerequisite for transforming differentiated cells into proliferative cancer cells [13].

**Fig. 1:**
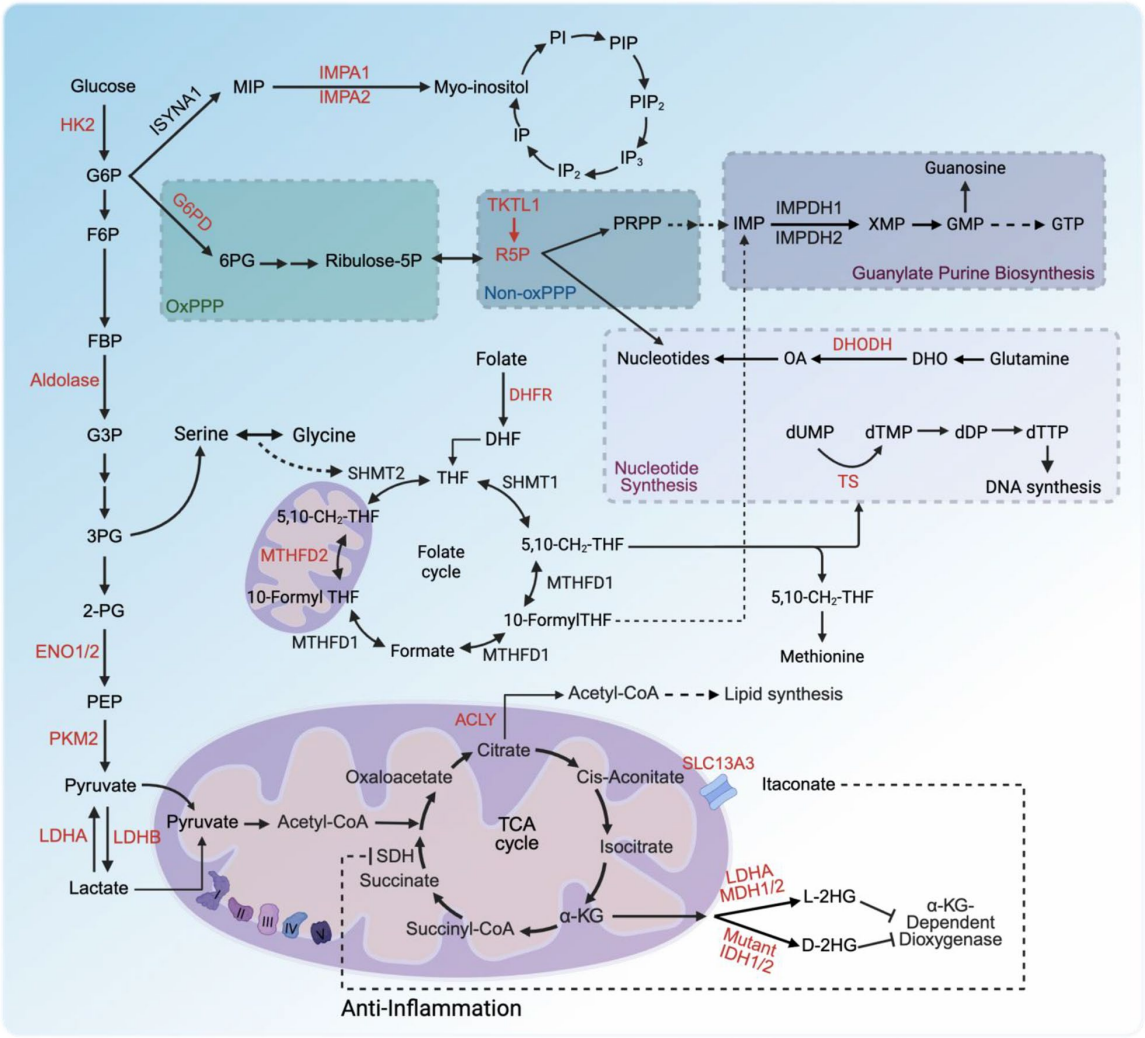
Glucose metabolism pathways and signaling metabolites. Glucose fuels glycolysis, the PPP, inositol biosynthesis, and the TCA cycle, supporting energy production, signal transduction, nucleotide synthesis, and epigenetic reprogramming. Metabolites like glucose, F6P, PEP, lactate, inositol, 2HG, and itaconate regulate target proteins, influencing tumor progression and immunity beyond their roles as precursors or energy sources. Enzymes such as aldolase, ENO1, LDHA/LDHB, IMPA1/2, ACOD1, and mutant IDH1/2 control signaling metabolite production, driving alternative biological functions in tumors and immunity

**Fig. 2:**
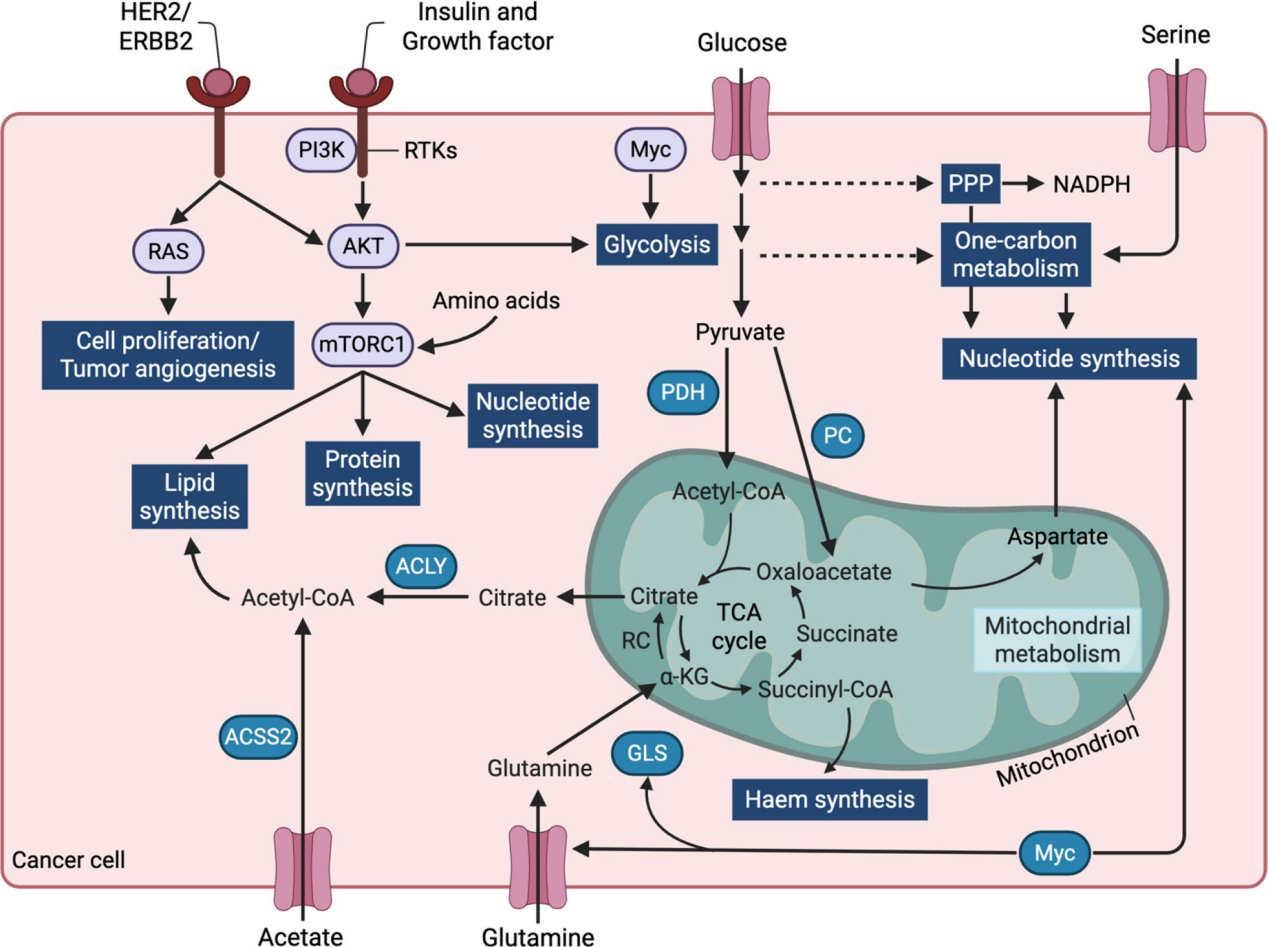
Regulatory Interactions in cancer metabolism. Metabolic pathways in cancer cells, regulated by insulin, growth factors, glucose, and serine. Insulin and growth factors activate PI3K, RTKs, AKT, and mTORC1, promoting lipid, protein, and nucleotide synthesis. Glucose is metabolized via glycolysis to pyruvate, which is converted to acetyl-CoA by PDH and PC, feeding the TCA cycle and supporting lipid synthesis via ACLY. Serine drives one-carbon metabolism, producing NADPH and nucleotides, regulated by PP. Glutamine supports the TCA cycle and haem synthesis via GLS, while acetate is converted to acetyl-CoA by ACSS2. MYC enhances glycolysis, mitochondrial metabolism, and haem synthesis, promoting cancer cell proliferation and survival. Arrows depict metabolic flux or regulatory interactions

Lactate, a key product of glycolysis, is converted from pyruvate by LDH. LDH has two isoforms, LDHA and LDHB. LDHA converts pyruvate into lactate and NAD^+^, while LDHB converts lactate into pyruvate, fueling oxidative metabolism. LDHA is essential for increased glycolysis and oncogenic functions in cancer cells and is a transcriptional target of the oncogene MYC, which provides molecular regulation for the Warburg effect [14]. Additionally, oncogenic transformation by RAS and SRC kinases enhances glucose uptake by upregulating glucose transporters [15]. Other proteins dysregulated in cancers and required for tumor growth, such as AKT, mTOR, and hypoxia-inducible factors (HIFs), individually promote glycolysis via transcriptional upregulation and phosphorylation of glucose transporters and glycolytic enzymes [16–18] (Figs. 1 and 2).

Oncogenic activation of RAS, c-MYC, or ERBB2 also upregulates HK2, which converts glucose to G6P and directly affects cellular glycolysis and glucose utilization in several in vitro and in vivo models [19, 20]. HK2 is also a transcriptional target of HIF-1α, which contributes to metabolic rewiring under hypoxic conditions [21, 22]. In a model of KRAS-driven lung cancer, HK2 is transcriptionally activated by the transcription factor BACH1, which is stabilized by antioxidant treatment, triggering metastatic spread favored by glycolytic metabolism in cancer cells [23]. As HK2 drives oncogenic functions and metabolic reprogramming in cancer cells to support growth and survival, targeting HK2 is a promising anti-tumor strategy. A well-characterized HK2 inhibitor, 2-deoxy-D-glucose (2-DG), competitively inhibits HK2 by mimicking glucose, reducing glucose-6-phosphate (G6P) production and suppressing tumor growth in various preclinical models [20]. A list of potential drugs targeting glucose metabolism is shown in Table 1.

**Table 1:** Summary of Potential Drugs Targeting Glucose Metabolism

Target	Drug name	Function	Mechanism	References
Glucose/Nsun2 binding	TAT-N28	Overcome anti-PD-L1 immunotherapy resistance in vivo	Disrupts glucose/NSUN2 binding, inhibits NSUN2 activity, triggers cGAS/STING-mediated innate immune response	[11]
HK2	2-Deoxy-D-glucose (2-DG)	Inhibits tumor growth	Competitive HK2 inhibitor, reduces G6P production and impairs glycolysis	[20]
G6PD	6-Aminonicotinamide	Enhances radiotherapy sensitivity	Inhibits G6PD, reduces NADPH levels	[27]
TKTL1	Oxythiamine	Inhibits tumor growth	Inhibits TKTL1, reduces R5P production	[34]
ACLY	Bempedonic Acid	Inhibits tumor growth in NSCLC and TNBC	Inhibits ACLY, disrupts lipid and nucleotide synthesis	[72]
Glutaminase	CB-839	Inhibits tumor growth in NSCLC and TNBC	Inhibits glutaminase, disrupts glutamine metabolism	[72, 73]
LDHA/LDH	Oxamate	Inhibits lactate dehydrogenase (LDH), critical for tumor growth	Pyruvate analog, non-selective LDH inhibitor (IC50 ~ 800 μM)	[105, 106]
LDHA/LDH	GSK2837808A	Potent LDHA inhibition, enhances T cell-mediated tumor killing	Selective LDHA inhibitor (IC50 = 2 nM), poor in vivo clearance	[83]
LDHA/LDH	GNE-140	Inhibits LDHA in glycolysis-dependent pancreatic cancer cells	Rewires metabolism to OXPHOS, lacks in vivo efficacy	[107]
LDHA/LDH	NCI-006	Inhibits LDHA, suppresses tumor growth	Pyrazole-based, nanomolar IC50, effective in MIA PaCa-2, HT29, Ewing sarcoma xenografts	[84–86]
LDHA/LDH) and mitochondrial complex I (OXPHOS)	NCI-006 + IACS-10759	Synergistic LDHA and mitochondrial complex I inhibition	Combines LDH inhibition with OXPHOS targeting, effective in vitro and in vivo	[87]
Aldolase	Aldometanib	Activates lysosomal AMPK, suppresses tumor growth in hepatocellular carcinom	Abolishes aldolase-FBP binding	[117–119]
ENO1/2	POMHEX	Inhibits ENO1/2, reduces PEP levels, suppresses tumor proliferation and metastasis	Inhibits ENO1/2 activity	[126]
IMPA1	Lithium Chloride	Inhibits IMPA1, reduces CRPC progression in combination with enzalutamide	Non-selective IMPA1 inhibitor, also inhibits GSK3, effective in CRPC xenografts	[110, 111]
D-2HG (Mutant IDH1/IDH2)	Ivosidenib	Inhibits mutant IDH1, reduces D-2HG production	Reverses D-2HG production, promotes AML differentiation (FDA-approved for AML)	[70, 71]
D-2HG (Mutant IDH1/IDH2)	Enasidenib	Inhibits mutant IDH2, reduces D-2HG production	Reverses D-2HG production, promotes AML differentiation (FDA-approved for AML)	[70, 71]
DHFR/TS	Methotrexate (MTX)	Inhibits DHFR, depletes folate cycle intermediates	Blocks thymidine/purine synthesis via TS inhibition, induces apoptosis	[30, 44–46]
Thymidylate synthase (TS)	5-Fluorouracil (5-FU)	Inhibits thymidylate synthase (TS), depletes dTTP	5-Fluoro-dUMP inhibits TS, may lead to dUMP accumulation	[47–50]
Thymidylate synthase (TS)	Pemetrexed	Inhibits TS, depletes dTTP	TS inhibition, dUMP-independent	[47–50]
MTHFD2	TH9619	Inhibits MTHFD2, reduces AML growth and metastasis	Potent (IC50 47 nM), inhibits AML xenograft growth	[33]
DHODH	Leflunomide/Teriflunomide	Inhibits DHODH, depletes (d)NTPs	Targets pyrimidine synthesis	[51–53]
DHODH	Brequinar	Inhibits DHODH, depletes (d)NTPs	Promising in clinical trials for pyrimidine synthesis inhibition	[51–53]
SLC13A3	2-(3-methylbenzyl) succinic acid	Induces ferroptosis resistance and sensitizes melanoma xenografts to ICB	Blocks itaconate binding to SLC13A3, inhibits NRF2-SLC7A11 axis, induces ferroptosis	[56]

Additionally, PKM2, which converts PEP to pyruvate in the glycolytic pathway, interacts with HIF-1α to increase the expression of its target genes, such as GLUT1 and LDHA, thus contributing to tumorigenesis [24, 25]. Collectively, cancer cells enhance glycolysis by upregulating the transcription of metabolic enzymes involved in glycolysis to sustain proliferation and survival (Figs. 1 and 2).

Beyond supporting proliferation, aerobic glycolysis shapes the TME by producing lactate, which fosters immunosuppression and therapy resistance [7]. This metabolic adaptation allows cancer cells to outcompete immune cells for glucose, impairing anti-tumor immunity while promoting tumor progression [9]. Collectively, cancer cells increase glycolysis by upregulating metabolic enzymes, such as HK2, PKM2, and LDHA, to sustain proliferation and survival. These insights into aerobic glycolysis elucidate its role in cancer biology and highlight opportunities for targeting glycolytic enzymes and their regulatory networks.

### Pentose phosphate pathway

The PPP is a critical metabolic route in cancer cells, providing NADPH for redox homeostasis and R5P for nucleotide biosynthesis [26]. NADPH, generated in the oxidative PPP, fuels reductive biosynthesis and neutralizes oxidative stress, supporting cancer cell survival and proliferation [27]. Glucose-6-phosphate dehydrogenase (G6PD), the rate-limiting enzyme, regulates NADPH/NADP + ratios and is implicated in tumorigenesis, particularly in KRAS-driven, LKB1-deficient lung cancer [28]. Additionally, metastatic melanoma cells rely heavily on NADPH from the folate pathway, highlighting NADPH production as a potential target to suppress metastasis [29].

Oncogenic signaling tightly controls the PPP. NRF2, activated by KEAP1 mutations, upregulates G6PD, phosphogluconate dehydrogenase (PGD), and transketolase (TKT), enhancing NADPH and R5P production [30]. MYC drives PPP gene expression to support nucleotide synthesis [31]. In hypoxic conditions, the non-oxidative PPP, mediated by transketolase-like 1 (TKTL1) and transaldolase (TALDO), modulates R5P output [32]. The PPP also interfaces with one-carbon metabolism via serine hydroxymethyltransferase (SHMT), producing 5,10-methylenetetrahydrofolate (5,10-CH2-THF) for methionine synthesis, with methylenetetrahydrofolate dehydrogenase 1/2 (MTHFD1/2) generating additional NADPH [30, 32] (Figs. 1 and 2).

Targeting PPP enzymes offers therapeutic promise. Inhibiting MTHFD2 with TH9619 induces thymidine depletion, suppressing acute myeloid leukemia (AML) growth [33]. G6PD inhibitors, such as 6-aminonicotinamide, reduce NADPH levels, enhancing radiotherapy sensitivity [27]. TKTL1 inhibitors like oxythiamine inhibit R5P production and tumor growth [34]. Combining PPP inhibitors with DNA-damaging agents, such as cisplatin, amplifies apoptosis [35]. However, challenges include the reliance of immune cells on NADPH, which may compromise anti-tumor immunity [8], and metabolic plasticity, enabling cancer cells to shift to glycolysis or glutamine metabolism. Dual inhibition of G6PD and HK2 has shown synergistic effects, suggesting a strategy to overcome resistance.

In summary, the PPP is a vital hub for cancer cell redox balance and biosynthesis, regulated by oncogenic signals and linked to one-carbon metabolism. Targeting enzymes like G6PD, MTHFD2, or TKTL1 holds therapeutic potential, but careful consideration of immune cell dependencies and metabolic adaptability is essential for effective and precise interventions.

### Nucleotide synthesis

One of the main outputs of one-carbon metabolism is biosynthesis of nucleotides, which provide building blocks (purines and pyrimidines) for DNA synthesis and subsequent cell proliferation. Pyrimidine and purine biosynthesis follows distinct biochemical pathways. In the pyrimidine de novo pathway, the base (orotate) is synthesized first and then attached to R5P via a phosphoribosyl pyrophosphate (PRPP)-dependent reaction catalyzed by uridine-monophosphate (UMP) synthase. In contrast, the purine de novo pathway begins with PRPP, and the aromatic base is constructed directly on the ribose scaffold through a series of enzymatic steps. Both pathways require ATP, glutamine-derived nitrogen, and aspartate as key substrates [34].

In the pyrimidine salvage pathway, (deoxy)nucleosides are phosphorylated to (d)NMPs in a single ATP-dependent step by enzymes such as uridine-cytidine kinase (UCK1/2), deoxycytidine kinase (DCK), or thymidine kinase (TK1/2) [35]. However, mammalian cells cannot efficiently salvage free pyrimidine bases (uracil, cytosine, thymine) via PRPP-dependent reactions or convert them back to orotate, rendering base salvage ineffective [36]. Purine salvage operates differently. Ribonucleosides (adenosine, guanosine, inosine) are first degraded to their respective bases (adenine, guanine, hypoxanthine) by purine nucleoside phosphorylase (PNP), then salvaged to NMPs via PRPP-dependent enzymes adenine phosphoribosyltransferase (APRT) and hypoxanthine–guanine phosphoribosyltransferase (HGPRT) [37, 38]. In contrast, purine deoxynucleosides (deoxyadenosine, deoxyguanosine) can be directly phosphorylated by DCK to form dAMP and dGMP, constituting the purine nucleoside–nucleobase salvage pathway [39].

Key oncogenes, including mutant KRAS [40], PI3K [41], and MYC [31], drive this elevation by upregulating de novo nucleotide synthesis enzymes and enhancing glucose uptake to fuel the production of ATP, ribose, and amino acid precursors. Conversely, loss of catabolic enzymes like SAMHD1, which degrades dNTPs to deoxynucleosides, increases nucleotide pools by limiting degradation [42]. The widespread elevation of nucleotide synthesis in cancer, its critical role in supporting malignancy, and the relative dispensability of de novo synthesis for most normal tissues make targeting de novo nucleotide metabolism a promising therapeutic strategy [34, 43] (Figs. 1 and 2).

Nucleotide synthesis enzymes are key drug targets in cancer therapy. Antifolates, like methotrexate (MTX), inhibit dihydrofolate reductase (DHFR) and deplete folate cycle intermediates critical for thymidine and purine synthesis, primarily via thymidylate synthase (TS) inhibition [30, 44, 45]. MTX’s efficacy spans cancer and autoimmune diseases, partly through apoptosis induction [46]. 5-Fluorouracil (5-FU) and pemetrexed target TS, causing dTTP depletion. 5-FU’s metabolite 5-fluoro-dUMP inhibits TS but can lead to dUMP accumulation, while pemetrexed’s TS inhibition is dUMP-independent [47–50]. As MTHFD2, which is overexpressed in cancers, promotes growth and metastasis, targeting MTHFD2 by its inhibitor TH9619 shows potent activity in inhibiting AML xenograft growth in vivo [33]. Dihydroorotate dehydrogenase (DHODH) inhibition, critical for pyrimidine synthesis, depletes (d)NTPs. leflunomide/teriflunomide and brequinar target DHODH, with brequinar showing promise in clinical trials [51–53].

### The tricarboxylic acid cycle

The TCA cycle, or Krebs cycle, serves as a pivotal metabolic hub, orchestrating energy production, biosynthesis, and signaling by integrating glucose-derived carbons in cancer and immune cells [54]. While the Warburg effect highlights glycolysis, many tumors harness the TCA cycle for OXPHOS and biosynthetic needs, often fueled by lactate as a primary carbon source in vivo [55]. This metabolic adaptability enables tumors to thrive under diverse nutrient conditions, supporting growth and metastasis [54]. Here, we explore the TCA cycle’s multifaceted roles in cancer metabolism, its biosynthetic outputs, and its profound influence on immune regulation in the TME.

Cancer cells boost glucose uptake via transporters (GLUT1/3), channeling pyruvate and lactate into the TCA cycle through pyruvate dehydrogenase (PDH) or pyruvate carboxylase (PC) [6, 56]. Lactate, imported via monocarboxylate transporters (MCTs), sustains TCA cycle activity and OXPHOS in tumors like non-small cell lung cancer (NSCLC) [55]. Glucose oxidation is the process by which glucose-derived carbons enter the TCA cycle and are oxidized to CO_2_, producing ATP through OXPHOS. While many cancers utilize aerobic glycolysis known as Warburg effect to meet their energy demand, certain cancer types such as NSCLC display substantial reliance on OXPHOS for ATP production, particularly in vivo, by utilizing lactate and/or other substrates to fuel the TCA cycle and subsequent electron transport chain (ETC) [55, 57, 58]. ETC can transfer electrons from NADH and FADH_2_ through complexes I–IV to oxygen, generating a proton gradient that drives ATP synthesis. Many cancer cells retain functional ETC activity to meet their energy and biosynthetic demands, especially under nutrient-limited or hypoxic conditions [59]. Oncogenic mutations like KRAS, MYC, AKT can increase electron flow through the ETC, enhancing ROS production and creating a feedback loop that sustains the cancer phenotype [60]. Excessive ROS can render cancer cells to adapt by upregulating antioxidant systems, maintaining ROS at a level that favors tumor growth without triggering cell death [60]. Hence, targeting ETC complexes could develop the potent therapeutic strategy for cancer therapy. Indeed, inhibition of ETC complexes like Complex I, II and III reduces cancer cell viability, tumor growth and overcomes chemotherapy resistance in various models [61, 62] (Figs. 1 and 2).

Oncogenes such as MYC and KRAS amplify TCA cycle enzymes, driving glutamine-derived α-KG production to fuel biosynthesis [31, 40]. α-KG, generated from isocitrate by isocitrate dehydrogenase (IDH) in the TCA cycle, is a key metabolic intermediate and signaling molecule. The TCA cycle powers biosynthesis essential for tumor proliferation. Citrate, exported via SLC25A1, is converted by ATP-citrate lyase (ACLY) into acetyl-CoA, fueling lipid synthesis for membrane assembly and histone acetylation for gene expression [54]. α-KG and oxaloacetate support amino acid synthesis (e.g., glutamate, glutamine), while aspartate drives nucleotide production, critical for DNA replication in KRAS-driven cancers [34, 40]. TCA cycle-derived NADH and NADPH bolster redox balance, mitigating oxidative stress [27].

The TCA cycle shapes immune dynamics in the TME. Tumor cells’ voracious consumption of glucose and glutamine starves effector T cells and M1 macrophages, limiting their TCA cycle activity and cytokine production [8, 9]. Conversely, Tregs and M2 macrophages, reliant on OXPHOS, flourish, fostering immunosuppression [63]. α-KG promotes Treg differentiation and M2 polarization, dampening Th1/Th17 responses [64]. Itaconate, produced by aconitase dehydrogenase (ACOD1), inhibits succinate dehydrogenase (SDH), curbing inflammation but suppressing CD8⁺ T cell activity [56, 65]. D-2HG and L-2HG further impair T cell, NK cell, and dendritic cell functions, enhancing Treg accumulation and immune evasion [66–69].

Targeting the TCA cycle holds therapeutic promise for cancer. FDA-approved IDH1/2 inhibitors (ivosidenib, enasidenib) reduce D-2HG levels, restore differentiation, and enhance anti-tumor immunity in AML [70, 71]. ACLY inhibitors (e.g., bempedonic acid) and glutaminase inhibitors (e.g., CB-839) disrupt lipid and nucleotide synthesis in NSCLC and triple-negative breast cancer [72, 73]. Blocking itaconate transport via SLC13A3 enhances immunotherapy efficacy [56]. Yet, challenges like metabolic plasticity and immune cell dependence on TCA cycle activity necessitate combining these inhibitors with PD-1/PD-L1 blockade and tailoring therapies to tissue-specific contexts [74].

### Glucose metabolism in immune regulation

Glucose metabolism is a critical determinant of immune cell function within the TME, where competition for nutrients shapes anti-tumor immunity. Cancer cells, through aerobic glycolysis (Warburg effect), consume large amounts of glucose, producing lactate and other metabolites that profoundly influence immune responses. This section explores how glucose metabolism regulate immune cell activation, differentiation, and effector functions, contributing to tumor immune evasion and therapeutic resistance.

#### Metabolic competition in the TME

Cancer cells upregulate glucose transporters (GLUT1/3) and glycolytic enzymes (e.g., HK2, LDHA) to fuel rapid proliferation, outcompeting immune cells for glucose in the nutrient-scarce TME [9]. Effector T cells, M1 macrophages, and DCs rely on glycolysis to support proliferation and cytokine production, whereas Tregs and M2 macrophages preferentially utilize OXPHOS [8]. This metabolic divergence creates a glucose-deprived environment that impairs effector immune cell functions while favoring immunosuppressive cells, promoting tumor progression [9].

#### One-carbon metabolism and immune evasion

One-carbon metabolism, linked to glucose via the PPP and serine synthesis, supports nucleotide biosynthesis and epigenetic regulation in both cancer and immune cells. The enzyme MTHFD2 drives the folate cycle, sustaining uridine diphosphate N-acetylglucosamine (UDP-GlcNAc) levels and cMYC O-GlcNAcylation, which upregulates PD-L1 expression, promoting immune evasion [75]. In immune cells, MTHFD2 is critical for T cell proliferation and cytokine production. In CD4⁺ T cells, MTHFD2 regulates Th17 function and Treg differentiation by maintaining purine biosynthetic intermediates, impacting FoxP3 expression [76]. MTHFD2 deficiency reduces disease severity in inflammatory models, suggesting its potential as a therapeutic target for autoimmune diseases, though its role in cancer immunity requires careful consideration due to immune cell dependency [76].

Similarly, inosine monophosphate dehydrogenase 2 (IMPDH2), a key enzyme in purine biosynthesis, is upregulated in cancers, promoting pro-tumorigenic phenotypes and chemotherapy resistance in glioblastoma, colorectal cancer, and triple-negative breast cancer [77–80]. However, IMPDH2 is also essential for T cell proliferation, as guanine nucleotides are critical for lymphocyte function [81]. Targeting folate or purine metabolism in the TME may thus compromise immune cell activity, necessitating strategies to balance tumor suppression with immune preservation.

Targeting glucose metabolism to modulate immune responses holds promise but faces challenges due to shared metabolic pathways between cancer and immune cells. Inhibiting GLUT1 sensitizes tumors to TNF-α-mediated cell death by elevating ROS, suggesting a strategy to enhance T cell-mediated killing [82]. LDHA inhibitors, such as GSK2837808A or NCI-006, reduce lactate production and tumor growth, but their impact on immune cell function requires further validation [83–87]. Combining metabolic inhibitors with immune checkpoint blockade (e.g., anti-PD-1/PD-L1) could alleviate TME immunosuppression, enhancing effector T cell activity. However, metabolic plasticity and tissue-specific effects necessitate tailored approaches to avoid impairing anti-tumor immunity.

## Part 2: glucose and its metabolites as signaling molecules

### Glucose as a signaling molecule

Glucose serves as a central core for building blocks in cellular metabolism due to its pivotal roles as both an energy source for glycolysis and TCA cycle and a precursor for biosynthetic pathways for purine and pyrimidine biosynthesis. In addition, glucose also directly acts as a signaling molecule by binding to specific proteins, initiating cascades that control tumor immunity, tumor progression and cell differentiation.

**Fig. 3:**
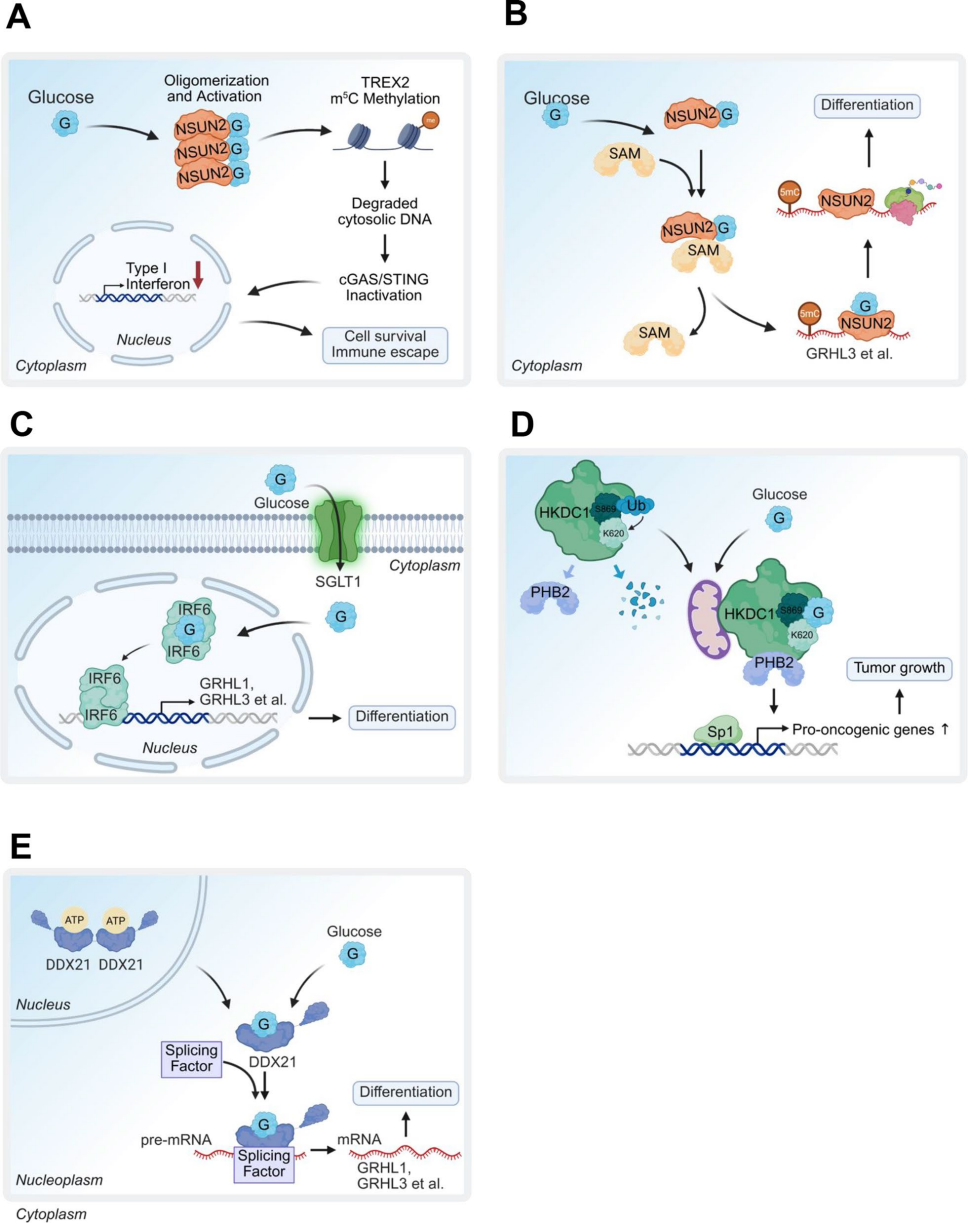
Glucose as a signaling molecule in cellular processes. **A** Glucose directly binds to NSUN2, enhancing its activity to promote TREX2-mediated cytosolic DNA degradation, resulting in cGas/STING pathway inactivation, which supports cell survival and immune evasion. **B** Glucose binds NSUN2, enhancing its interaction with S-adenosyl-L-methionine to boost m5C methylation and translation of pro-differentiation mRNAs like GRHL3, promoting keratinocyte differentiation and epidermal development. **C** Glucose binds to IRF6, promoting its dimerization and nuclear translocation, which activates transcription of target genes such as GRHL3, essential for epidermal differentiation. **D** Glucose stabilizes HKDC1, enabling it to sequester prohibitin 2 (PHB2) and suppress Sp1-driven oncogenic gene expression, inhibiting tumor growth. **E** Glucose interacts with DDX21, forming a DDX21 monomer that translocates from the nucleus to the nucleoplasm, triggering RNA splicing to promote cell differentiation in progenitor cells

Glucose can function as a cofactor to directly bind to and activate NSUN2, an RNA 5-methylcytosine (m5C) methyltransferase triggering m5C RNA methylation of TREX2, leading to stabilizing TREX2 exonuclease to restrict cytosolic DNA accumulation for cGAS/STING activation and subsequent type-I interferon-mediated apoptosis and CD8^+^ T cell infiltration in TME for promoting tumorigenesis and PD-L1 immunotherapy resistance [11] (Fig. 3A). In addition, glucose binds to NSUN2 and promotes its activation by enhancing its interaction with S-adenosyl-L-methionine (SAM) leading to promoting mRNA translation critical for epidermal differentiation. This interaction promotes the proximity of NSUN2 to the translation machinery, thus facilitating global mRNA translation [88] (Fig. 3B). Moreover, glucose also regulates epidermal differentiation by modulating interferon regulatory factor 6 (IRF6). Glucose binds directly to IRF6 and facilitates its dimerization and nuclear translocation resulting in the expression of its target genes such as GRHL3, critical for keratinocyte differentiation. This glucose-dependent activation of IRF6 highlights a non-metabolic role for glucose in orchestrating tissue specific differentiation programs [89] (Fig. 3C).

Hexokinase domain-containing 1 (HKDC1) has also been reported to serve as a glucose sensor. Upon glucose binding, HKDC1 sequestrates PHB2 to repress oncogenic transcription factor Sp1-driven expression of genes linked to tumor growth and survival [90] (Fig. 3D). Another study points out that HKDC1 promotes immune evasion in a CD8^+^ T cell-dependent manner by upregulating STAT1/PD-L1 signaling [91]. In keratinocytes, glucose influences differentiation via the RNA-binding protein DDX21. Glucose binds to DDX21’s ATP-binding domain, converting dimers into monomers that translocate to the nucleoplasm [12]. There, DDX21 binds SCUGSDGC RNA motifs, modulating mRNA splicing of oncogenic genes like GRHL3. Additionally, DDX21 senses dsRNA within the DDX1-DDX21-DHX36 complex, triggering IFN-β production via TRIF, or inhibits RIG-I-mediated IFN-β signaling [92] (Fig. 3E). However, the role of glucose in these immune-related functions remains unstudied [12].

While glucose itself can play the role as a signaling metabolite to modulate innate immune response in the TME, targeting direct glucose sensor can be promising therapeutic strategies to overcome immunotherapy resistance. Given NSUN2 is identified as a direct glucose sensor whose activation by glucose drive tumorigenesis and immunotherapy resistance [11], blocking the binding between NSUN2 and glucose would be the potential therapeutic strategy for impeding tumorigenesis and overcoming immunotherapy resistance. Indeed, TAT-N28 peptide has been identified to abolish glucose/NSUN2 binding and NSUN2 activation [11], suggesting that TAT-N28 peptide is a potential small-molecule drug for overcoming glucose/NSUN2/TREX2 axis in driving anti-PD-L1 immunotherapy resistance via cGAS/STING repression. It will be of interest to explore the in vivo efficiency of the TAT-N28 peptide in suppressing tumorigenesis and overcoming anti-PD-L1 immunotherapy resistance.

### Metabolites generated from glucose metabolism serve as signaling molecules

Beyond the production of the energy and building blocks for cell proliferation and survival, recent studies indicate that glucose metabolism also generates numerous intermediate metabolites that can function as signaling metabolites to directly impact signaling and epigenetics for cancer and immune cell regulation. In this section, we highlight some key metabolites derived from glucose metabolism that function as signaling metabolites orchestrate various biological processes. The metabolites including lactate, inositol, FBP, PEP, α-KG, 2HG, and itaconate were selected and highlighted in this section because of their direct roles as signaling molecules that bind specific protein targets for regulating epigenetic modifications and/or modulating the immune responses in the TME, cancer progression and therapeutic resistance. These metabolites exemplify how glucose metabolism extends beyond energy and biosynthesis to orchestrate complex biological processes. Other glucose-derived metabolites, such as pyruvate, acetyl-CoA, and nicotinamide adenine dinucleotide (NAD^+^), which can also act as signaling molecules to affect nuclear and cellular functions, are discussed briefly in a dedicated subsection below.

### Lactate

Lactate, a primary product of glycolysis, is not only a metabolic byproduct but also a critical signaling metabolite that orchestrates diverse biological functions in cancer and immune regulation. Lactate is a key regulator of immune responses. High lactate levels in the TME, driven by LDHA activity, foster immunosuppression by multiple mechanisms. Lactate uptake by immune cells via MCTs activates pathways that skew immune cell differentiation. For instance, lactate drives naïve CD4⁺ T cells toward Treg differentiation, enhancing immunosuppression by upregulating FoxP3 expression through inhibition of ATP5B-mediated mTOR phosphorylation and HIF-1α synthesis [93, 94]. In CD8⁺ T cells, lactic acid (the protonated form of lactate) disrupts TCA cycle dynamics by favoring PDH over PC, reducing cytotoxic activity [95].

**Fig. 4:**
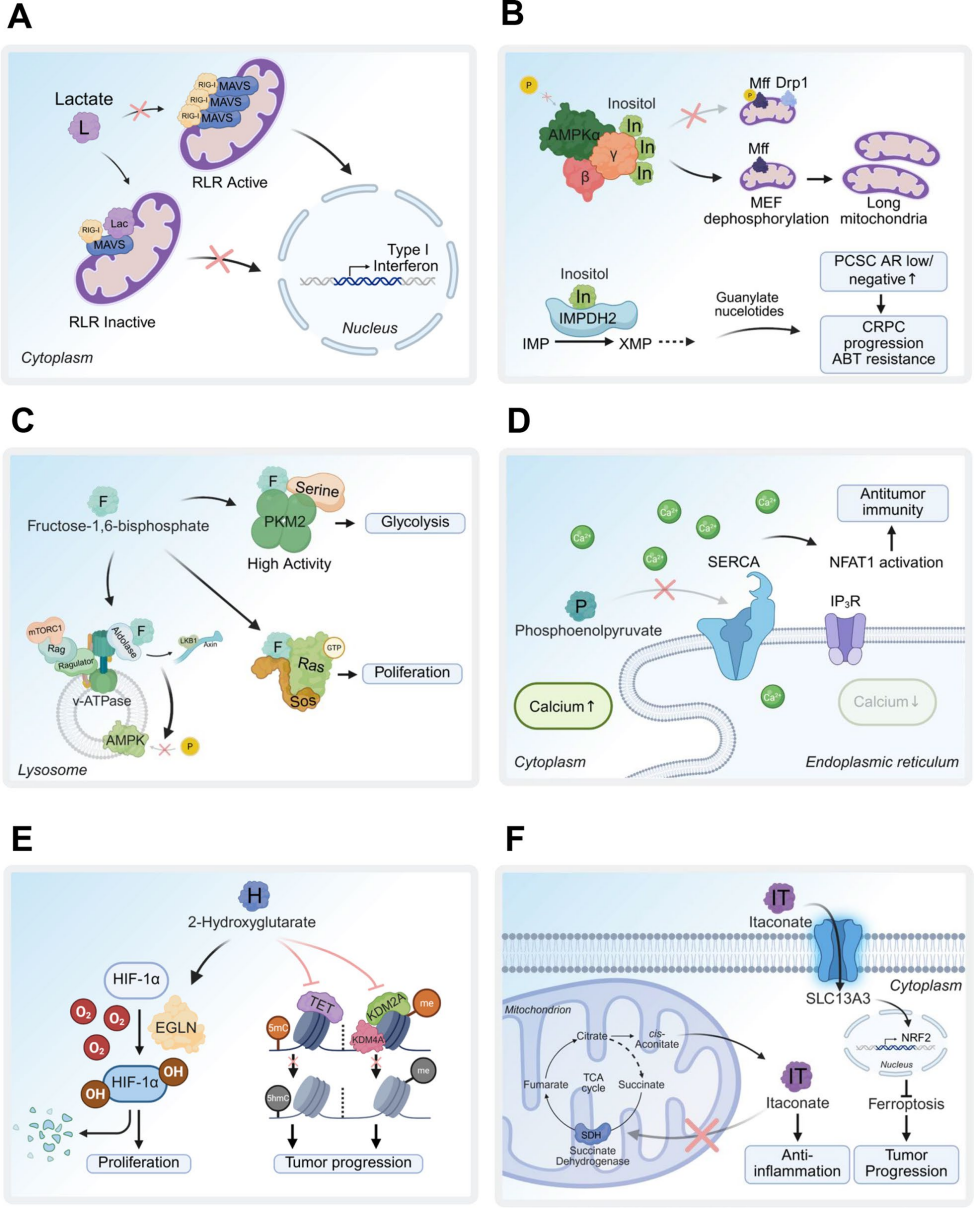
Signaling metabolites and their protein targets in diverse biological functions. **A** Lactate binds to RIG-I/MAVS, inhibiting MAVS aggregation and mitochondrial localization, which suppresses RLR signaling and IFN production, promoting viral infection. **B** Inositol binds to the AMPKg subunit, inactivating AMPK and inhibiting mitochondrial fission. In prostate cancer stem cells, inositol activates IMPDH2 to enhance guanylate purine biosynthesis, driving CRPC progression and ABT resistance. **C** FBP disrupts the AXIN/LKB1-v-ATPase/Ragulator interaction by targeting aldolase, leading to AMPK inactivation. FBP also activates PKM2 to boost glycolysis flux and, in yeast, binds to the Sos/Ras complex to promote Ras-GTP activation and oncogenic Ras functions. **D** PEP associates with SERCA, inhibiting its activity to block calcium reuptake, enhancing effector T cell functions and antitumor immunity in the TME. **E** 2HG activates prolyl hydroxylase (EGLN), triggering HIF-1a degradation to promote cell proliferation. It also inhibits TET activity, reducing 5-methylcytosine (5mC) conversion to 5-hydroxymethylcytosine (5hmC), and suppresses KDM2A/KDM4A-mediated histone demethylation, contributing to tumor progression. **F** Itaconate binds to and inhibits SDH, impairing the TCA cycle and reducing inflammatory cytokine production in activated macrophages

Lactate also modulates macrophage polarization. Tumor-derived lactate promotes M2-like macrophage phenotypes via the HIF-1α-VEGF and ATP6V0d2-HIF-2α-VEGF pathways, enhancing pro-tumorigenic functions [7, 96]. In glioblastoma, LDHA activates the ERK-YAP1/STAT3 axis, upregulating CCL2 and CCL7 to recruit macrophages, which in turn deliver LDHA to tumor cells via extracellular vesicles, forming a tumor-macrophage symbiotic loop that drives progression [97]. Our study demonstrates that glycolysis-derived lactate inhibits RIG-I-like receptor (RLR) signaling by directly binding to mitochondrial antiviral-signaling protein (MAVS), suppressing type-I interferon (IFN) production (Fig. 4A) [98]. This interaction suggests a therapeutic opportunity to enhance anti-tumor and antiviral immunity by disrupting the lactate-MAVS axis.

In the TME, lactate accumulation under hypoxic or inflammatory conditions promotes histone and protein lactylation, modulating gene expression and protein function [99]. Specifically, lactate-driven lactylation of NBS1 at lysine 388 and MRE11 at lysine 673 enhances homologous recombination (HR)-mediated DNA repair by facilitating MRE11–RAD50–NBS1 (MRN) complex formation and recruitment of HR repair proteins to DNA double-strand break sites, contributing to chemotherapy resistance [100, 101]. These findings highlight lactate’s role in driving therapy resistance and immune modulation, as detailed in recent reviews [102–104].

Targeting lactate metabolism, particularly LDHA, offers therapeutic potential. In K-Ras- and EGFR-driven lung cancer mouse models, *Ldha* knockout inhibits tumor growth [105]. Early LDHA inhibitors like oxamate, a pyruvate analog, lack selectivity and require high IC50 (~ 800 μM) [105, 106]. In contrast, GSK2837808A potently inhibits LDHA (IC50 ~ 2 nM) with 10- to 80-fold selectivity over LDHB in liver and breast cancer cell lines, though its in vivo efficacy is limited by poor clearance [83]. GNE-140, another potent LDHA inhibitor, shows efficacy in glycolysis-dependent pancreatic cancer cell lines but lacks in vivo activity [107]. NCI-006, a pyrazole-based LDHA inhibitor, achieves nanomolar IC50, favorable pharmacokinetics, and in vivo efficacy in xenograft models [84–86]. Combining NCI-006 with IACS-10759, a mitochondrial complex I inhibitor, synergistically enhances therapeutic effects by counteracting oxidative rewiring, as demonstrated in vitro and in vivo [87]. Despite these advances, the in vivo efficacy of LDHA inhibitors requires further validation to overcome challenges like metabolic plasticity and off-target effects.

Collectively, lactate’s dual role as an energy source and signaling metabolite underscores its significance in cancer progression and immune suppression. Developing selective LDHA inhibitors with robust in vivo efficacy remains a promising strategy for cancer therapy.

### Inositol

Inositol, derived from G6P through glycolysis, is synthesized via a two-step process involving inositol-3-phosphate synthase 1 (ISYNA1), which converts G6P to myo-inositol-3-phosphate, followed by dephosphorylation by inositol monophosphatase (IMPA1) to produce inositol. Alternatively, inositol can be directly imported via the sodium/myo-inositol cotransporter (SLC5A3). Inositol serves as a critical precursor for the phosphatidylinositol (PI) cycle, generating phosphatidylinositol phosphates (PIPs) and inositol phosphates (IPs), which are essential for cell membrane trafficking, calcium homeostasis, and cell survival [108]. In cancer, inositol metabolism is implicated in tumor progression, with high ISYNA1 expression linked to poor survival in colon adenocarcinoma, suggesting its potential as a prognostic marker [109].

Recent studies highlight inositol’s role as a signaling metabolite beyond its precursor functions. In prostate cancer stem cells (PCSCs), IMPA1 upregulation increases inositol levels, which directly bind and activate inosine monophosphate dehydrogenase 2 (IMPDH2), a key enzyme in purine biosynthesis. This interaction sustains PCSC stemness and drives castration-resistant prostate cancer (CRPC) progression, contributing to androgen ablation therapy (ABT) resistance [110]. Pharmacological inhibition of IMPA1 with lithium chloride, combined with enzalutamide, significantly reduces CRPC progression in vivo in CRPC patient-derived xenograft (PDX) and castrated TRAMP mouse models [110]. However, lithium chloride’s non-specificity, as it also inhibits glycogen synthase kinase 3 (GSK3) [111], underscores the need for more selective IMPA1 inhibitors.

Inositol also acts through a signaling metabolite to regulates mitochondrial dynamics and energy homeostasis. Loss of IMPA1 or stress-induced inositol depletion triggers AMP-activated protein kinase (AMPK)-mediated mitochondrial fission, impairing mitochondrial function [112]. Mechanistically, inositol binds to the AMPKγ subunit, suppressing AMPK activation and preventing excessive mitochondrial fission (Fig. 4B). The AMP/inositol ratio, alongside the AMP/ATP ratio, is a critical determinant of AMPK activation under energy stress, positioning inositol as a key regulator of cellular energy balance [112]. These findings suggest that targeting IMPA1-mediated inositol signaling could be a promising therapeutic strategy for CRPC and likely other cancers, provided more specific inhibitors are developed.

### Fructose-1,6-bisphosphate

FBP, a key glycolytic intermediate, is produced from fructose-6-phosphate by phosphofructokinase-1 (PFK1) and plays a significant role in cancer metabolism beyond its metabolic function. FBP acts as a signaling metabolite, influencing oncogenic pathways and cellular processes. In yeast, FBP binds to the H-RAS/SOS1 complex, a guanine nucleotide exchange factor (GEF), promoting RAS-GTP activation and enhancing oncogenic signaling, which suggests that increased glycolytic flux may amplify tumor-promoting pathways [113]. Additionally, FBP modulates protein kinase activity. It binds to aldolases, disrupting the lysosomal complex of v-ATPase, Ragulator, AXIN, LKB1, and AMPK, leading to AMPK inactivation [114]. This positions aldolases as FBP sensors that regulate AMPK activity during glucose deprivation. FBP, along with serine (derived from the glycolytic intermediate 3-phosphoglycerate), also acts as an allosteric activator of PKM2, enhancing glycolysis to support cancer cell proliferation [115].

High glucose uptake and elevated glycolytic flux are hallmarks of cancer metabolic reprogramming, making imbalanced glycolysis a potential therapeutic target. Accumulation of FBP due to aldolase A (ALDOA) deletion induces an energy-consuming state, disrupting glycolytic balance and reducing cancer cell proliferation in hepatocellular carcinoma models, thereby extending survival [116]. The aldolase inhibitor aldometanib blocks FBP binding to aldolase, selectively activating lysosomal AMPK and exerting tumor-suppressive effects, particularly in hepatocellular carcinoma, where AMPK plays a critical anti-tumor role [117–119] (Fig. 4C). These findings highlight the therapeutic potential of targeting FBP-mediated signaling by inhibiting aldolase to disrupt cancer metabolism and growth, though further validation in diverse cancer models is needed to confirm efficacy and specificity.

### Phosphoenolpyruvate

PEP, a high-energy glycolytic intermediate, is generated from 2-phosphoglycerate (2PG) by α-enolase 1 (ENO1). PEP serves as a critical metabolite for energy production and supports biosynthetic pathways, including nucleotide, lipid, and amino acid synthesis through metabolic branching. Beyond its metabolic role, ENO1 exhibits oncogenic functions, acting as a plasminogen receptor on tumor cell surfaces to promote cancer cell proliferation, migration, invasion, and metastasis [120]. ENO1 is overexpressed in various cancers, and its upregulation drives tumorigenesis and metastasis by modulating the AMPK/mTOR signaling pathway [121, 122]. In immune cells, ENO1 is essential for T cell effector functions, regulating T cell activation and the suppressive activity of induced Tregs [123, 124].

As a signaling metabolite, PEP modulates anti-tumor immunity in the TME. PEP inhibits sarco/endoplasmic reticulum Ca^2^⁺-ATPase (SERCA), preventing endoplasmic reticulum (ER) Ca^2^⁺ reuptake. This sustains Ca^2^⁺-NFAT signaling, enhancing T cell activation and effector functions critical for anti-tumor responses [125] (Fig. 4D). Given ENO1’s oncogenic roles and high expression in cancers, it is a promising therapeutic target. The ENO1/2 inhibitor POMHEX, a potent prodrug, demonstrates anti-neoplastic activity in orthotopic brain tumor models and is well-tolerated in primates, suggesting a favorable pharmacological profile for human use [126]. However, ENO1 inhibition may reduce PEP levels in the TME, potentially impairing effector T cell functions. Thus, the therapeutic efficacy of ENO1 inhibitors, such as POMHEX, requires further validation in immunocompetent mouse models to balance tumor suppression with immune preservation.

### α –ketoglutarate and 2-hydroxyglutarate

α-KG, generated from isocitrate by IDH in the TCA cycle, is a key metabolic intermediate and signaling molecule. As a cofactor for dioxygenases, including TET and JMJD enzymes, α-KG promotes epigenetic modifications that drive cell differentiation, acting as a tumor suppressor in various cancers [127, 128]. However, in aggressive tumors, α-KG supports glutamine metabolism, fueling biosynthetic demands and tumor growth [127]. In the immune system, α-KG enhances regulatory T cell (Treg) differentiation and M2 macrophage polarization, suppressing Th1/Th17 responses and inflammation, thus contributing to an immunosuppressive TME [63, 64].

However, mutations in TCA cycle enzymes, such as isocitrate dehydrogenase (IDH1/2), SDH, or fumarate hydratase (FH), generate oncometabolites like D-2-hydroxyglutarate (D-2HG), succinate, and fumarate, which disrupt epigenetic regulation and promote tumorigenesis in gliomas, AML, and renal cancers [10, 129–138]. Mutations in IDH1 or IDH2, prevalent in gliomas [131, 132], AML [133–136], chondrosarcomas [137], and cholangiocarcinomas [138], confer neomorphic activity [129], converting α-KG to the oncometabolite D-2-hydroxyglutarate (D-2HG) [74]. However, α-KG can also be the alternative substrate for LDH and malate dehydrogenase (MDH) to produce L-2HG under physiological condition like hypoxia [139]. Numerous studies have shown that accumulation of D/L-2HG exerts oncogenic activity. D/L-2HG has been shown to suppress α-KG-dependent dioxygenases activity for epigenetic modifications and gene expression, thereby modulating cell fate including tumor progression [140, 141]. D/L-2HG displays the inhibition of ten-eleven translocation (TET) family 5-methylcytosine (5mC) hydrolases activity that converts 5mC to 5-hydroxylmethycytosine (5hmC) and human JHDM1A/KDM2A or KDM4A demethylase activity [67, 142, 143] (Fig. 4E). Moreover, D/L-2HG occupies the same space as α-KG does in the active site of histone demethylases, indicating that D/L-2HG is a competitive inhibitor of α-KG for histone demethylases [142]. In addition, D-2HG, but not L-2HG, reduces HIF levels by stimulating HIF prolyl 4-hydroxylases (EGLN) activity, which enhances the proliferation and soft agar growth of human astrocytes [144].

In the TME, both D-2HG and L-2HG suppress CD8⁺ T cell and NK cell functions while promoting Treg accumulation, creating an immunosuppressive environment [66–68]. In addition, in TME, L-2HG is accumulated in pancreatic cancer cells, serum samples from pancreatic cancer patients due to increased LDH or MDH activity under hypoxia condition [69]. Secreted L-2HG inhibits T cell proliferation and migration in pancreatic cancer, thereby suppressing anti-tumor immunity [69]. L-2HG, produced under hypoxia by LDH or MDH, accumulates in pancreatic cancer, impairing dioxygenase and electron transport chain functions [141, 145]. L-2HG dehydrogenase (L2HGDH) regulates its levels, and its overexpression curbs renal tumorigenesis [145]. The tissue-specific effects of these oncometabolites underscore the need to understand contextual genetic and environmental drivers of cancer [140, 146].

Collectively, these finding provides new insights into that development of therapeutic approaches to reduce oncometabolites, D-2HG and L-2HG, can achieve not only the inhibition of intrinsic tumor growth but also the enhancement of immune cell antitumor activity. Therapeutically, FDA-approved inhibitors ivosidenib (IDH1) and enasidenib (IDH2) reduce D-2HG levels, restore differentiation, and enhance anti-tumor immunity in AML, demonstrating clinical efficacy [70, 71]. These findings highlight the potential of targeting 2HG production to inhibit tumor growth and boost immune responses, although tissue-specific effects and combination strategies warrant further exploration.

### Itaconate

Itaconate, a metabolite derived from the TCA cycle, is produced in activated macrophages through the decarboxylation of cis-aconitate by ACOD1, also known as immune-responsive gene 1 (IRG1). Itaconate exerts potent anti-inflammatory effects by inhibiting NLRP3 inflammasome activation, thereby reducing IL-1β and TNF production [147]. Interestingly, itaconate as an anti-inflammatory metabolite directly alkylates cysteine residues 151, 257, 288, 273 and 297 on the protein KEAP1, enabling NRF2 to increase the expression of downstream target genes consisting of anti-oxidant and anti-inflammatory capacities like *Hmox1* or glutathione (GHS) [148]. IRG/itaconate-mediated NRF2 expression enhances macrophage phagocytosis leading to improved outcomes in intracerebral hemorrhagic stroke and peritonitis [149–151]. In mouse models of sepsis, itaconate and its derivative, 4-octyl itaconate (4-OI), inhibits STING phosphorylation and the production of downstream inflammatory factors like IFN-β and TNF-α through alkylation of STING at cysteine sites 65, 71, 88, and 147 to ameliorate inflammation [152]. Itaconate also modulates macrophage activation by inhibiting SDH, reducing succinate oxidation, mitochondrial respiration, and inflammatory cytokine production [65]. This is achieved through its direct inhibition of SDH activity in vitro and in vivo to block succinate oxidation, which could lead to reduction of mitochondrial respiration, and inflammatory cytokine production during macrophage activation [65] (Fig. 4F).

In the TME, itaconate plays a complex role in tumor biology and anti-tumor immunity. *ACOD1*-deficient macrophages suppress tumor growth and enhance the efficacy of PD-1/PD-L1 blockade [153]. Itaconate as an immune checkpoint metabolite secreted from myeloid-derived suppressor cells (MDSCs) to suppress CD8^+^ T cell functions [154]. Therefore, targeting ACOD1 enables to promote antitumor immunity and enhance the efficacy of immune checkpoint blockade in cancer. Another potential target to block the inhibitory effects of itaconate on antitumor immunity is SLC13A3, which is identified as itaconate transporter for tumor cells [56]. Itaconate import via SLC13A3 transporter promotes tumor progression via activating NRF2-SLC7A11 axis to induce ferroptosis resistance [56] (Fig. 4F). Additionally, SLC13A3-mediated itaconate uptake by tumor cells directly alkylates PD-L1 at cysteine 272 to promote PD-L1 stabilization leading to tumor immune evasion [155]. As such, SLC13A3 inhibition enhances the efficacy of anti-CTLA-4 immune checkpoint blockade, thus providing a potential strategy to overcome immune suppression in the TME [155]. It is important to note that SLC13A3 inhibitor, 2-(3-methylbenzyl) succinic acid, is able to overcomes ICB resistance and sensitizes xenograft melanoma tumor to ferroptosis in vivo [56]. These findings highlight itaconate’s dual role in inflammation and tumor immunity, underscoring the need for targeted therapies to modulate its effects in the TME while preserving anti-tumor immune responses.

### Other glucose-derived metabolites as signaling molecules

Beyond the above metabolites, glucose metabolism also generates additional signaling molecules like pyruvate, acetyl-CoA, and NAD^+^ that influence cellular compartments, including the nucleus and mitochondria. Pyruvate, a key glycolytic product, regulates histone deacetylases (HDACs) by acting as a substrate for PDH, generating acetyl-CoA for histone acetylation and gene expression [54]. Acetyl-CoA, produced via pyruvate dehydrogenase (PDH) or ATP citrate lyase (ACLY), serves as a critical cofactor for histone acetyltransferases (HATs), linking glucose metabolism to epigenetic regulation and gene transcription in cancer cells [54]. NAD^+^, generated through glycolysis and the TCA cycle, acts as a cofactor for sirtuins and poly(ADP-ribose) polymerases (PARPs), hence modulating DNA repair, gene expression, and stress responses [2]. These metabolites signal to the nucleus and other cellular compartments, integrating metabolic status with cellular functions. However, their roles are often context-dependent and intertwined with broader metabolic networks, highlighting the need for further investigation in cancer-specific contexts.

## Conclusion and perspective

**Fig. 5:**
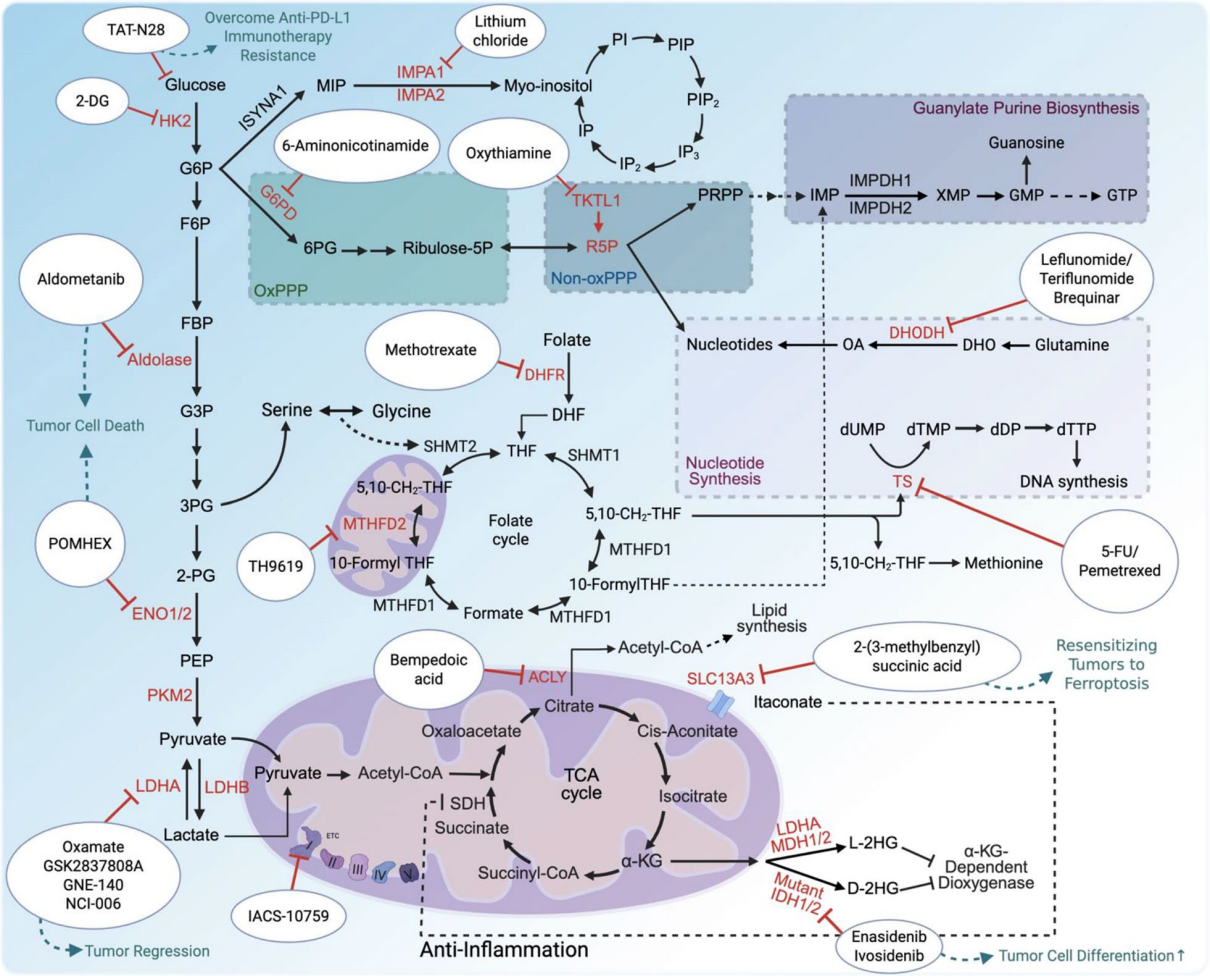
Potential metabolism-based drugs for blocking the actions of signaling metabolites. TAT-N28 disrupts the binding between glucose and NSUN2 to inhibit NSUN2 activity leading to triggering cGas/STING-mediated innate immune response for overcoming anti-PD-L1 immunotherapy resistance. 2-DG competitively inhibits HK2 by mimicking glucose, reducing G6P production and suppressing tumor growth. Aldometanib, an aldolase inhibitor, activates lysosomal AMPK activity by disrupting the binding between FBP and aldolase, displaying inhibition of tumor growth. POMEX, a prodrug for targeting enolase, has shown anti-neoplastic activity. LDH/LDHA inhibitors like oxamate, GSK2837808A, GNNE-140 and NCI-160 have revealed the inhibition of tumor growth in vitro and in vivo. 2-(3-methylbenzyl) succinic acid blocking itaconate binding to SLC13A3 transporter inhibits NRF2-SLC7A11 axis to drive ferroptosis leading to tumor regression. Ivosidenib and Enasidenib both are mutant IDH1/2 inhibitors to reduce oncometabolite 2HG levels, thus controling epigenetic reprogramming for T cell functions and tumor differentiation

Glucose metabolism is a cornerstone of cancer biology, driving energy production, biosynthetic demands, and signaling cascades that orchestrate tumor progression and immune modulation. This review highlights how glucose, through its metabolic pathways and direct sensing mechanisms, fuels cancer cell proliferation, shapes the TME, and influences immune responses. Beyond serving as a source of ATP and biosynthetic precursors, glucose and glucose-derived metabolite, such as lactate, inositol, FBP, PEP, itaconate, α-KG, and 2HG, function as signaling molecules that regulate signaling and epigenetic modifications to orchestrate cancer progression, immune evasion, and therapy resistance. These insights underscore the multifaceted roles of glucose in cancer and open new avenues for therapeutic intervention [1, 2] (Fig. 5).

Despite significant advances, several challenges persist. The tissue-specific effects of metabolic mutations, such as those in IDH1/2, suggest that tumor initiation and progression depend on the interplay of genetic alterations, tissue context, and environmental factors [74]. For instance, co-mutations in KRAS and STK11 drive distinct metabolic vulnerabilities in lung cancer (e.g., reliance on pyrimidine metabolism and OXPHOS compared to pancreatic ductal adenocarcinoma, where serine metabolism and DNA methylation are prioritized [156–158]. These differences highlight the need for precision medicine approaches that integrate cancer genetics, tissue origin, and dietary influences. Isotope tracing has elucidated how glucose and lactate fuel the TCA cycle, but it falls short of explaining how tumor genotypes and tissue contexts shape metabolic profiles [55, 159, 160].

A key challenge in targeting cancer metabolism is achieving specificity for malignant cells while sparing normal tissues. Metabolic pathways, such as glycolysis and nucleotide synthesis, are shared by cancer and immune cells, complicating therapeutic strategies [8]. For example, inhibiting LDHA may suppress tumor growth but risks impairing immune cell function, as seen with ENO1 inhibitors potentially reducing PEP levels critical for T cell activation [125, 126]. Species-specific differences further complicate translation from preclinical models to humans, as evidenced by LDHA mutations causing hemolytic anemia in mice but not humans [161, 162]. These findings emphasize the importance of rigorous in vivo validation using immunocompetent models and careful consideration of off-target effects.

Progress in drug development is essential to address these challenges. Metabolic inhibitors like ivosidenib and enasidenib, FDA-approved for IDH1/2-mutant AML, demonstrate the potential of targeting oncometabolites [70, 71]. Techniques such as structural biology, pharmacokinetics, and live imaging, including ^18^F-glutamine PET, can improve drug specificity and verify target engagement [163]. Inhibitors with low-nanomolar IC50 values are optimal for reducing side effects, but high tumor protein expression may require innovative strategies like proteolysis-targeting chimeras (PROTACs) [164]. However, PROTAC-based metabolic drugs face obstacles, including optimization difficulties, the hook effect, limited E3 ligase ligand diversity, low permeability and solubility, and toxicity concerns [165–167]. Intriguingly, a promising prodrug-based chemical endocytic medicinal chemistry strategy enhancing PROTAC binding to CD36 has shown improved anti-tumor efficacy by increasing permeability, solubility, and efficacy [168]. Exploiting the CD36 pathway for PROTAC development in glucose metabolism targeting could revitalize poorly absorbed PROTACs for clinical applications.

Looking forward, integrating cancer metabolism with genomics, immune profiling, and real-time metabolic imaging will enable personalized therapies. Combining metabolic inhibitors with immunotherapies, such as PD-1/PD-L1 blockade, could enhance anti-tumor immunity by alleviating TME immunosuppression. Addressing metabolic plasticity, tissue specificity, and immune cell dependencies will be critical to translate these strategies into effective and safe treatments. By leveraging these insights, the field can move toward precision cancer therapies that exploit metabolic vulnerabilities while preserving host immunity.

## Data Availability

No datasets were generated or analysed during the current study.

## Abbreviations

2HG: 2-Hydroxyglutarate
5-FU: 5-Fluorouracil
5hmC: 5-Hydroxymethylcytosine
5mC: 5-Methylcytosine
ACLY: ATP-Citrate Lyase
ACOD1: Aconitase Dehydrogenase
ALDOA: Aldolase A
AML: Acute Myeloid Leukemia
AMPK: AMP-Activated Protein Kinase
APRT: Adenine Phosphoribosyltransferase
ATP: Adenosine Triphosphate
BACH1: BTB Domain and CNC Homolog 1
CCL2: Chemokine (C–C Motif) Ligand 2
CCL7: Chemokine (C–C Motif) Ligand 7
cGAS: Cyclic GMP-AMP Synthase
CRPC: Castration-Resistant Prostate Cancer
D-2HG: D-2-Hydroxyglutarate
L-2HG: L-2-Hydroxyglutarate
DCK: Deoxycytidine Kinase
DCs: Dendritic Cells
DDX1: DEAD-Box Helicase 1
DDX21: DEAD-Box Helicase 21
DHF: Dihydrofolate
DHFR: Dihydrofolate Reductase
DHODH: Dihydroorotate Dehydrogenase
DHX36: DEAH-Box Helicase 36
DNA: Deoxyribonucleic Acid
dNTP: Deoxynucleotide Triphosphate
EGLN: HIF prolyl 4-hydroxylases
ENO1: α-Enolase 1
ER: Endoplasmic Reticulum
ERK: Extracellular Signal-Regulated Kinas
FBP: Fructose-1,6-Bisphosphate
FH: Fumarate Hydratase
G6P: Glucose-6-Phosphate
G6PD: Glucose-6-Phosphate Dehydrogenase
GEF: Guanine Nucleotide Exchange Factor
GLUT1: Glucose Transporter 1
GLUT3: Glucose Transporter 3
GRHL3: Grainyhead-Like Transcription Factor 3
GSK3: Glycogen Synthase Kinase 3
HATs: Histone Acetyltransferases
HDACs: Histone Deacetylases
HGPRT: Hypoxanthine–Guanine Phosphoribosyltransferase
HIF-1α: Hypoxia-Inducible Factor-1α
HK2: Hexokinase 2
HKDC1: Hexokinase Domain-Containing 1
HR: Homologous Recombination
ICB: Immune Checkpoint Blockade
IDH1: Isocitrate Dehydrogenase 1
IDH2: Isocitrate Dehydrogenase 2
IFN-β: Interferon-β
IMPA1: Inositol Monophosphatase 1
IMPDH2: Inosine Monophosphate Dehydrogenase 2
IPs: Inositol Phosphates
IRF6: Interferon Regulatory Factor 6
IRG1: Immune-Responsive Gene 1
ISYNA1: Inositol-3-Phosphate Synthase 1
JHDM1A: JmjC Domain-Containing Histone Demethylase 1A
KDM2A: Lysine Demethylase 2A
KDM4A: Lysine Demethylase 4A
KEAP1: Kelch-Like ECH-Associated Protein 1
L-2HG: L-2-Hydroxyglutarate
L2HGDH: L-2HG Dehydrogenase
LDH: Lactate Dehydrogenase
LDHA: Lactate Dehydrogenase A
LDHB: Lactate Dehydrogenase B
MAVS: Mitochondrial Antiviral-Signaling Protein
MCTs: Monocarboxylate Transporters
MDH: Malate Dehydrogenase
ME1: Malic Enzyme 1
5,10-CH2-THF: 5,10-Methylenetetrahydrofolate
MRN: MRE11–RAD50–NBS1
mTOR: Mammalian Target of Rapamycin
MTHFD1: Methylene Tetrahydrofolate Dehydrogenase 1
MTHFD2: Methylene Tetrahydrofolate Dehydrogenase 2
MTX: Methotrexate
NAD^+^: Nicotinamide Adenine Dinucleotide (Oxidized)
NADH: Nicotinamide Adenine Dinucleotide (Reduced)
NADPH: Nicotinamide Adenine Dinucleotide Phosphate (Reduced)
NBS1: Nibrin
NFAT: Nuclear Factor of Activated T-Cells
NK: Natural Killer
NMPs: Nucleoside Monophosphates
NSCLC: Non-Small Cell Lung Cancer
NSUN2: RNA 5-Methylcytosine Methyltransferase
OXPHOS: Oxidative Phosphorylation
PARPs: Poly(ADP-Ribose) Polymerases
PC: Pyruvate Carboxylase
PCSCs: Prostate Cancer Stem Cells
PD-1: Programmed Cell Death Protein 1
PDH: Pyruvate dehydrogenase
PDXs: Patient-Derived Xenografts
PDH: Pyruvate Dehydrogenase
PD-L1: Programmed Death-Ligand 1
PEP: Phosphoenolpyruvate
PFK1: Phosphofructokinase-1
PHB2: Prohibitin 2
PI: Phosphatidylinositol
PIPs: Phosphatidylinositol Phosphates
PKM2: Pyruvate Kinase M2
PNP: Purine Nucleoside Phosphorylase
PPP: Pentose Phosphate Pathway
PRPP: Phosphoribosyl Pyrophosphate
PROTACs: Proteolysis-Targeting Chimeras
R5P: Ribose-5-Phosphate
RIG-I: Retinoic Acid-Inducible Gene I
RLR: RIG-I-Like Receptor
RNR: Ribonucleotide Reductase
ROS: Reactive Oxygen Species
RTKs: Receptor Tyrosine Kinases
SAM: S-adenosyl-L-methionine
SDH: Succinate Dehydrogenase
SERCA: Sarco/ER Ca2 + -ATPase
SHMT: Serine Hydroxymethyltransferase
SLC13A3: Solute Carrier Family 13 Member 3
SLC25A1: Solute Carrier Family 25 Member 1
SLC5A3: Sodium/Myo-Inositol Cotransporter
STAT1: Signal Transducer and Activator of Transcription 1
STING: Stimulator of Interferon Genes
TALDO: Transaldolase
TCA: Tricarboxylic Acid
TET: Ten-Eleven Translocation
TGF-β: Transforming Growth Factor-β
THF: Tetrahydrofolate
TK1/2: Thymidine Kinase ½
TKT: Transketolase
TME: Tumor Microenvironment
TNF-α: Tumor Necrosis Factor-α
TRAMP: Transgenic Adenocarcinoma of the Mouse Prostate
Tregs: Regulatory T Cells
TRIF: TIR-Domain-Containing Adapter-Inducing Interferon-β
TS: Thymidylate Synthase
UCK1/2: Uridine-Cytidine Kinase 1/2
UDP-GlcNAc: Uridine Diphosphate N-Acetylglucosamine
UMP: Uridine Monophosphate
VEGF: Vascular Endothelial Growth Factor
YAP1: Yes-Associated Protein 1
